# Independent and Interactive Influences of Environmental UVR, Vitamin D Levels, and Folate Variant *MTHFD1*-rs2236225 on Homocysteine Levels

**DOI:** 10.3390/nu12051455

**Published:** 2020-05-18

**Authors:** Patrice Jones, Mark Lucock, Charlotte Martin, Rohith Thota, Manohar Garg, Zoe Yates, Christopher J. Scarlett, Martin Veysey, Emma Beckett

**Affiliations:** 1 School of Environmental & Life Sciences, University of Newcastle, Ourimbah, NSW 2258, Australia; Mark.Lucock@newcastle.edu.au (M.L.); charlottemartin1981@gmail.com (C.M.); c.scarlett@newcastle.edu.au (C.J.S.); Emma.Beckett@newcastle.edu.au (E.B.); 2 Hunter Medical Research Institute, New Lambton Heights, NSW 2305, Australia; 3 Nutraceuticals Research Group, University of Newcastle, Callaghan, NSW 2308, Australia; R.thota@massey.ac.nz (R.T.); manohar.garg@newcastle.edu.au (M.G.); 4 Riddet Institute, Massey University, Palmerston North 4442, New Zealand; 5 Biomedical Sciences & Pharmacy, University of Newcastle, Callaghan, NSW 2308, Australia; Zoe.Yates@newcastle.edu.au; 6 Hull-York Medical School, University of Hull, Hull YO10 5DD, UK; martin.veysey@hyms.ac.uk

**Keywords:** homocysteine, folate, vitamin D, ultraviolet radiation, genetic variant

## Abstract

Elevated homocysteine (Hcy) levels are a risk factor for vascular diseases. Recently, increases in ultraviolet radiation (UVR) have been linked to decreased Hcy levels. This relationship may be mediated by the status of UVR-responsive vitamins, vitamin D and folate, and/or genetic variants influencing their levels; however, this has yet to be examined. Therefore, the independent and interactive influences of environmental UVR, vitamin D and folate levels and related genetic variants on Hcy levels were examined in an elderly Australian cohort (*n* = 619). Red blood cell folate, 25-hydroxyvitamin D (25(OH)D), and plasma Hcy levels were determined, and genotyping for 21 folate and vitamin D-related variants was performed. Erythemal dose rate accumulated over six-weeks (6W-EDR) and four-months (4M-EDR) prior to clinics were calculated as a measure of environmental UVR. Multivariate analyses found interactions between 6W-EDR and 25(OH)D levels (p_interaction_ = 0.002), and 4M-EDR and *MTHFD1*-rs2236225 (p_interaction_ = 0.006) in predicting Hcy levels. The association between 6W-EDR and Hcy levels was found only in subjects within lower 25(OH)D quartiles (<33.26 ng/mL), with the association between 4M-EDR and Hcy occurring only in subjects carrying the *MTHFD1*-rs2236225 variant. 4M-EDR, 6W-EDR, and *MTHFD1*-rs2236225 were also independent predictors of Hcy. Findings highlight nutrient–environment and gene–environment interactions that could influence the risk of Hcy-related outcomes.

## 1. Introduction

Homocysteine (Hcy) is a non-essential amino acid produced as an intermediate product in the synthesis of methionine and cysteine. Elevated levels of Hcy (i.e., hyperhomocysteinemia) can lead to the onset of multiple cardiovascular and neurovascular outcomes, such as atherosclerosis, stroke, and Alzheimer’s disease [1,2]. Several genetic, dietary, and other lifestyle factors are established determinants of Hcy status. The effects of these determinants are commonly examined in isolation; however, interactions may exist between determinants that lead to variation in Hcy levels and related risk of disease, and this is understudied. Investigation into such environment–gene–nutrient interactions in Hcy metabolism is limited but may offer new avenues in modifying Hcy and its related risk of pathology.

An inverse relationship between folate and Hcy is well established due to the central role of folate in converting Hcy to methionine [3,4]. Several environmental and genetic factors that regulate systemic folate status, such as dietary intake of folate, or the *MTHFR*-rs1801133 (commonly *MTHFR*-C677T) variant are recognised as key modulators of Hcy status. An emerging determinant of blood folate status—ultraviolet radiation (UVR)—is also a potential modulator of Hcy. UVR exposure has been shown to cause the breakdown of folates in vitro [5,6], and increases in UVR exposure have been associated with a decreased folate status in several human cohort studies [6,7,8,9,10]. More recently, we have reported that increases in environmental UVR levels are also associated with decreases in Hcy levels [11]. This association is counter-intuitive given the well-known inverse relationship between folate and Hcy levels, but it may be modulated in part by folate-related genetic factors, which require further investigation. Previous investigation into the negative association between UVR levels and red blood cell folate levels have found that this association was dependent on the presence of the *MTHFR*-rs1801133 variant. Furthermore, the distribution of multiple folate-related polymorphisms are linked to latitude [12] and skin phototype [13], indicating multiple folate polymorphisms that may interact with UVR exposure and potentially modulate the relationship between UVR and Hcy levels.

The relationship between Hcy and UVR may in part be dictated by changes in vitamin D levels. The relationship between UVR environment and vitamin D levels is well known, with vitamin D levels also having been reported to be inversely associated with Hcy. An inverse association between blood vitamin D and Hcy was first shown in data from the National Health and Nutrition Examination Survey (2001–2006) [14] and has since been replicated in several observational and intervention studies examining both healthy subjects [15,16] and subjects with Hcy-related conditions [17,18]. The mechanism by which vitamin D is linked to Hcy has not been elucidated, but likely relates to the influences of vitamin D on cystathionine B-synthase activity, a key enzyme in the folate-independent transsulfuration pathway that interconverts cysteine and Hcy [19]. However this relationship has only been demonstrated in vitro [19]. Hcy may be influenced by changes in vitamin D levels, as well as variants in related genes that influence overall vitamin D function. Multiple genes control vitamin D synthesis, metabolism and activity, with the frequency of multiple common variants in these genes known to vary by UVR environment and latitude [20,21,22,23,24,25].

Therefore, the aim of the present study was to further examine the negative association between environmental UVR and Hcy status reported in an elderly Australian cohort [26], examining whether this association is modulated by independent or interactive influences of vitamin D and/or folate levels, and related genetic factors.

## 2. Materials and Methods

### 2.1. Subjects

This study was a secondary analysis using samples and pre-existing data from the Retirement Health and Lifestyle Study (RHLS), a cross-sectional study examining the health and lifestyle of older Australians (>65 years) living in the Central Coast region of NSW, Australia (*n* = 650) [11,26,27,28]. This Australian elderly cohort was an appropriate focus for this investigation and previous studies due to Australians, in general, being exposed to high levels of environmental UVR [29], with elderly Australians a sub-population particularly at risk of adverse UVR-related effects as biological photoprotective factors reduce with age [30]. Briefly, subjects were eligible to be included in the initial RHLS if they were ≥65 years and lived independently within the community or resided in retirement villages on the Central Coast, NSW, Australia for at least the last 12 months. Subjects were ineligible if they did not live independently, if another household member was already taking part in the study, if they had language/communicative difficulties and/or were unable to provide informed consent. Subjects were eligible to be included in this secondary analysis if data was available for plasma Hcy levels (*n* = 619). Subjects provided written informed consent, and ethics approval for the study was obtained from the University of Newcastle Human Research Ethics Committee (reference no. H-2008-0431). Further details on the original study population and design have been reported previously [11,26,27,28].

### 2.2. Blood Biochemistry; Hcy and Vitamin Levels

Fasting blood samples of subjects were collected in EDTA-lined tubes. Blood samples were stored at −20 °C as whole blood, or stored at −80 °C as plasma or serum components in lithium heparin tubes or tubes containing clot activator respectively. Red blood cell (RBC) folate, serum vitamin B_12_ and creatinine were assessed by the Hunter Area Pathology Service via standardised assays. Total plasma Hcy was measured by a selective fluorescence assay (JD Biotech Corp, Taipei, Taiwan; linear range 1–60 umol/L, CV < 8%) [31,32]. All samples were tested in triplicate, and control samples were tested between batches as per manufacturer’s protocol (JD Biotech Corp, Taipei, Taiwan). Vitamin D levels were assessed via HPLC methods as the total serum concentration of 25-hydroxyvitamin D (25(OH)D) [33]. The detection range for this method was 9–193 ng/mL with a CV% < 10%. Method precision and accuracy were examined for quality control via repetitive assessments of triplicates of quality control samples prepared with different concentrations of vitamin D (20 nmol/L and 40 nmol/L 25(OH)D).

### 2.3. Dietary Intake, Smoking Status, and Body Mass Index

Dietary intake of subjects was estimated using a validated self-administered food frequency questionnaire (FFQ) covering 225 food items and all food groups, with questionnaires analysed using Foodworks^TM^ software (V.2.10.146; Xyris Software, Brisbane, QLD, Australia). These food frequency questionnaires were as published in previous studies [28,31] and were adapted from the Commonwealth Scientific and Industrial Research Organisation (CSIRO) FFQ [34]. Additional intake of nutrients via supplement use was included in calculated nutrient intakes as total dietary equivalents when applicable. Subject dietary data was not used if FFQs were invalid, with FFQs considered invalid if they were incomplete, or subjects reported excess (>30,000 kj/day) or deficient (<3500 kj/day) energy consumption, or excessive consumption of a single food group (≥11 serves/day) [28,31].

The smoking status of subjects was evaluated via an interviewer-administered questionnaire and subjects were categorised as non-smokers, ex-smokers or current smokers. Only 3% of subjects were current smokers, and therefore current smokers and those with a history of smoking were combined in the analyses. Subject’s body mass index (BMI (kg/m^2^)) was calculated using anthropometric measures taken during clinic appointments via standard procedures [27], and subjects were classified as underweight (BMI; <18.5), normal weight (BMI; 18.5–24.9), overweight (BMI; 25–29.9), or obese (BMI > 30). Only 2% of subjects were classified as underweight and were considered with those of normal BMIs in analyses.

### 2.4. Genotyping of Vitamin D and Folate Genetic Variants

**Figure 1:**
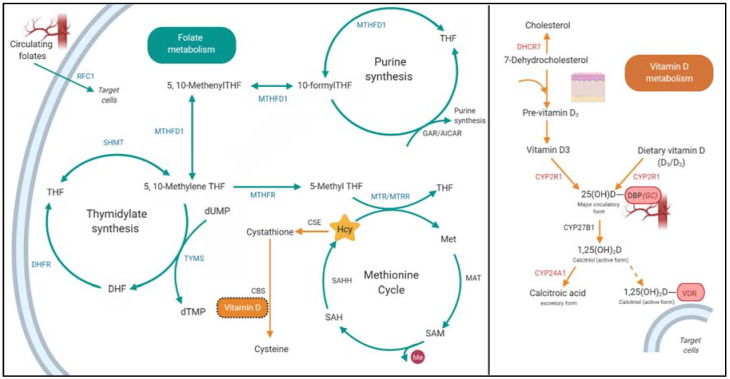
Examined genetic variants involved in folate and vitamin D metabolic pathways. Examined folate-related genetic variants reside within key genes coding for the enzymes (blue) involved in three interconnected pathways which support activities of folate in the methionine cycle, which allows for regulation homocysteine, and pathways related to the synthesis of thymidylate (thymine precursor) and purines. These pathways are interconnected as they rely on the same pool of reduced folates within the cell, so disruption to any of these pathways via genetic variation can lead to changes in Hcy levels [4]. RFC1 is a carrier protein which transports circulating folates into target cells. Hcy may be regulated through an alternative folate-independent process—the transsulfuration pathway. Hcy is converted to cysteine in this pathway via CSE and CBS. Vitamin D is previously shown to influence CBS activity [19], and this is the suggested mechanism, explaining previous reports of Hcy and vitamin D levels being inversely related [15,16]. Examined vitamin D-related genetic variants reside within genes coding for the key enzymes (red) involved in vitamin D synthesis and metabolism, which may influence vitamin D levels and/or activity and therefore have the potential to influence vitamin D’s role in influencing Hcy levels. Abbreviations: Folate metabolism; AICAR, 5-aminoimidazole-4-carboxamide ribonucleotide; CBS, cystathionine beta-synthase; CSE, Cystathionine gamma-lyase; DHF, dihydrofolate; DHFR, dihydrofolate reductase; dTMP, deoxyuridine monophosphate; dUMP, deoxyuridine monophosphate; GAR, glycinamide ribonucleotide; Hcy, homocysteine; MAT, adenosylmethionine synthetase; Met, methionine; MTHFD1, methylenetetrahydrofolate dehydrogenase 1; MTHFR, 5,10-methyleneTHF reductase; MTR, methionine synthase; MTRR, methionine synthase reductase; RFC1, reduced folate carrier gene; SAH, S-adenosyl hcy; SAHH, S-adenosylhomocysteine hydrolase; SAM, S-adenosyl methionine; SHMT, serinehydroxymethyl transferase; THF, tetrahydrofolate; TYMS, thymidylate synthase. Vitamin D metabolism; CYP24A1, 24-hydroxylase; CYP27B1, 1α-hydroxylase; CYP2R1, 25-hydroxylase; DBP/GC, vitamin D-binding protein; DHCR7, 7-dehydrocholesterol reductase; VDR, vitamin D receptor. Figure created with BioRender.

Genomic DNA was isolated from whole blood samples using Qiagen QIAamp mini-kits following manufacturer’s protocols for blood samples (Qiagen, Hilden, Germany). Genotypes were assessed for 10 folate-related genetic variants (*MTRR*-rs1801394, *MTR*-rs1805087, *MTHFR*-rs1801133, *MTHFR*-rs1801131, *SHMT*-rs1979277, *MTHFD1*-rs2236225, *RFC1*-rs1051266, *TYMS*-rs11280056, *TYMS*-rs45445694, and *DHFR*-rs70991108) and 11 vitamin D-related variants (*GC*-rs4588, *CYP2R1*-rs10741657, *DHCR7/NADSYN1*-rs12785878, *CYP24A1*-rs17216707, *VDR*-rs4516035, *VDR*-rs757343, *VDR*-rs2228570, *VDR*-rs731236, *VDR*-rs7975232, *VDR*-rs11568820, and *VDR*-rs1544410). These variants were selected for assessment as they are common and well-characterized variants related to changes in vitamin status and activity (Figure 1).

Genotyping was undertaken via mixed PCR methods. The majority of variants were genotyped following previously outlined RFLP-PCR methods [35,36,37,38,39,40,41,42,43,44,45,46,47]; *MTRR*-rs1801394, *MTR*-rs1805087, *MTHFR*-rs1801133, *MTHFR*-rs1801131, *SHMT*-rs1979277 *MTHFD1*-rs2236225, *RFC1*-rs1051266, *TYMS*-rs11280056, *GC*-rs4588, *CYP2R1*-rs10741657, *VDR*-rs4516035, *VDR*-rs757343, *VDR*-rs2228570, *VDR*-rs731236, *VDR*-rs7975232, and *VDR*-rs1544410. Variants *DHFR*-rs70991108 and *VDR*-rs11568820 were assessed following allele-specific PCR [48,49], with *TYMS*-rs45445694 evaluated via PCR and gel electrophoresis [50]. Genotyping for *DHCR7/NADSYN1*-rs12785878 and *CYP24A1*-rs17216707 were performed through qPCR methods using Taqman genotyping assays (Assay IDs; C_32063037_10 and C_33659702_10).

### 2.5. Estimation of Environmental UVR Levels: Accumulated Area Erythemal Dose Rate

Coordinates of subjects’ reported residential location were used to gather data on erythemal dose rate (EDR) in subject location prior to clinic appointments, gathered from NASA’s Total Ozone Mapping Spectrometer (Accessed via https://giovanni.gsfc.nasa.gov/giovanni/). EDR is a measure of the potential for biological damage to be caused by UVR [51]. Information was gathered for the total amount of EDR accumulated over two time periods of interest—over the six weeks (6W-EDR) and four months (4M-EDR) prior to the subject’s clinic appointments. 4M-EDR was assessed in a previous investigation examining relationships between environmental UVR and Hcy and/or RBC folate levels [11], and was chosen due to four months being the approximate lifespan of an RBC (i.e., relevant to RBC folate turnover), respectively. 6W-EDR was also assessed in the current study, with six weeks being indicative of the time-lag between UVR exposure and serum 25(OH)D changes [52,53]. EDR values ranged considerably between RHLS subjects and captured varying seasons due to original subject recruitments and clinic appointments spanning > 18 months (>80 clinic dates). Further details on the method have been published previously [11,54,55].

### 2.6. Statistical Analyses

Statistical analyses were performed using JMP software (V.14.2.0; SAS Institute Inc., Cary, NC, USA). Descriptive statistics (means, 95% confidence intervals, ranges, and frequencies) were calculated and presented as appropriate. Multifactorial modelling (standard least squares regression) was used to examine associations between variables of interest, with multiplicative interaction terms included in models where appropriate. Adjustment variables included age, sex, and key determinants of Hcy levels—dietary intake of vitamin B_6_ and alcohol, RBC folate, vitamin B_12_ and creatinine levels, BMI category, and smoking status [56,57,58]. Mixed direction stepwise regression was undertaken using significance levels of *p* ≤ 0.250 or *p* > 0.250 to enter or remove variables from models where appropriate. Mallow’s Cp criterion was used for selecting the model where Cp first approaches *p* variables. Multiple comparisons of least-squares means were made using Tukey’s HSD post hoc tests. Adjusted R^2^ values and *p*-values are reported for final models, with standardised parameter estimates (β) and *p*-values reported for individual variables. Significance level (*p* < 0.05) was adjusted using the Bonferroni method [59] to account for multiple testing where appropriate.

## 3. Results

### 3.1. Subject Characteristics

The mean age of subjects was 77 years (Table 1), with 56% being female. Mean RBC folate, 25(OH)D and Hcy levels were within the reference ranges, with reference ranges defined as 317–1422 nmol/L for RBC folate [60] and 5–15 μmol/L for Hcy levels [61]. The mean 25(OH)D level was higher than the range considered adequate (20–28 ng/mL) in the Australian population [62]. However, 14% of the cohort had 25(OH)D levels indicative of vitamin D deficiency (<20 ng/mL) [62]. Furthermore, 16% of the cohort had mild elevations in Hcy levels (15–35 μmol/L); however Hcy levels beyond the reference range for Hcy (5–15 μmol/L) are expected in elderly populations [61]. There were no incidences of folate deficiency (RBC folate < 317 nmol/L).

Reported intake of alcohol, creatinine levels, vitamin B_12_ levels, and smoking status differed by sex. Mean reported alcohol intake and creatinine levels were higher in males compared to females (10.4 (95% CI; 9.0–11.4) vs. 4.4 (3.7–5.1); *p* < 0.001, and 10.6 (10.0–11.2) vs. 8.4 (7.8–8.9); *p* < 0.001). Vitamin B_12_ levels were lower in males 224.6 (207.4–241.8) vs. 251.0 (235.6–266.2); *p* = 0.03). More males had a history of smoking (64% vs. 32%; *p* < 0.001).

The frequencies of examined folate and vitamin D genetic variants in this cohort are outlined in Table S1. Genotype frequencies did not deviate from Hardy–Weinberg expectations, except for the *GC*-rs4588 variant (Table S1). Genotypes were further classified as binary variables, with analyses comparing presence vs. absence of the polymorphic allele. *DHCR7*/*NADSYN1*-rs12785878 and *VDR*-rs7975232 variants were excluded from further analysis due to uneven groups, with >95% of subjects carrying polymorphic alleles for these variants.

### 3.2. Independent and Interactive Influences of EDR and Levels of 25(OH)D and RBC Folate on Hcy Levels

Increases in EDR accumulated over six weeks prior to clinic appointment (6W-EDR) was associated with decreased Hcy levels (unadjusted model; β = −0.25, *p* < 0.001; Table 2). A similar negative association was also shown between 4M-EDR and Hcy levels as previously reported [11] (unadjusted model; β = −0.29, *p* < 0.001; Table 2). Both these associations remained following adjustments with 25(OH)D and RBC folate levels (β = −0.24, *p* < 0.001 and β = −0.29, *p* < 0.001; Model 1, Table 2), and following further adjustments with potential confounders (β = −0.24, *p* < 0.001 and β = −0.30, *p* < 0.001; Model 2, Table 2). Notably, neither 25(OH)D or RBC folate levels were significant independent predictors of Hcy levels in these models (Table 2; Models 1–2) or when the binary associations between Hcy and either 25(OH)D or RBC folate were considered (Figure S1).

Potential environment–nutrient interactions between EDR measures and levels of 25(OH)D and/or folate in determining Hcy levels were examined to consider if the effects of EDR measures were dependent on levels of UVR-sensitive vitamins. A significant interaction between 6M-EDR and 25(OH)D in predicting Hcy was observed (unadjusted model; p_interaction_ = 0.01; Table 3). This interaction remained following adjustments with potential Hcy determinants and when considering multiple testing corrections using the Bonferroni method (Adjusted model; p_interaction_ = 0.002; Table 2). The direct effect of 6W-EDR was also an independent predictor of Hcy levels in unadjusted and adjusted models (β = −0.24, *p* < 0.001 in both cases; Table 3). When stratifying the association between 6W-EDR and Hcy by quartiles of 25(OH)D levels, this association was only significant for subjects in the two lower quartiles of 25(OH)D levels (Q1; β = −0.35, *p* = 0.001, Q2; β = −0.35, *p* = 0.001; Table 4). However, the overall interaction between 6W-EDR and 25(OH)D quartiles (when assessed as a categorical variable) did not remain when applying strict adjustments to the *p*-value threshold via the Bonferroni method (p_interaction_ = 0.004; Table 4).

### 3.3. Independent and Interactive Influences of Vitamin D and/or Folate Genetic Variants on Hcy Levels

Stepwise regression for all examined vitamin D and folate genetic variants resulted in two folate-related variants being entered into the predictive model for Hcy (*DHFR*-rs70991108 and *MTHFD1*-rs2236225). Neither variant was found to be a significant independent predictor of Hcy levels in unadjusted and adjusted models (Table S2).

Potential interactions between these variants and RBC folate and/or EDR measures were also assessed to consider if influences of EDR or folate were modified by *DHFR*-rs70991108 and *MTHFD1*-rs2236225 variants. No significant interactions were observed between variants and RBC folate in predicting Hcy. However, *MTHFD1*-rs2236225 was a significant independent predictor of Hcy levels following consideration of interactions with RBC folate (β = −0.11, *p* = 0.02; adjusted model; Table S3). Presence of *MTHFD1*-rs2236225 polymorphic allele was associated with higher Hcy levels relative to ancestral homozygotes (10.7 μmol/L (95% CI; 8.9–12.6 μmol/L) vs. 9.3 μmol/L (7.4–11.3 μmol/L).

A significant interaction was found between *MTHFD1*-rs2236225 and 4M-EDR in predicting Hcy levels (adjusted models; p_interaction_ = 0.006; Table 5). *MTHFD1*-rs2236225, 4M-EDR, and 6M-EDR were also direct independent predictors of Hcy levels in these models (adjusted models; Table 5). When the association between 4M-EDR and Hcy levels was stratified by carriage of the *MTHFD1*-rs2236225 polymorphic allele, a significant association was only observed in subjects carrying the polymorphic allele (β = −0.28, *p* < 0.001).

Significant interactions identified between EDR measures, 25(OH)D levels, and/or *MTHFD1*-rs2236225 in previous analyses were considered together in multivariate models. When considering interactions between 6W-EDR and both 25(OH)D levels and *MTHFD1*-rs2236225 in a single multivariate model, only the interaction between 6W-EDR and 25(OH)D levels were significant (p_interaction_ ≤ 0.001; adjusted model; Table 6). The direct effect of 6W-EDR was also a significant predictor of Hcy levels in this model (β = −0.19, *p* = 0.001), with the overall model predicting 9% of variance in Hcy levels. When considering interactions between 4M-EDR and both 25(OH)D levels and *MTHFD1*-rs2236225, only the interaction between 4M-EDR and *MTHFD1*-rs2236225 remained significant (p_interaction_ = 0.006; adjusted model; Table 6). The direct effects of *MTHFD1*-rs2236225 and 4M-EDR were also significantly associated with Hcy levels in this model (β = −0.09, *p* = 0.04 and β = −0.21, *p* ≤ 0.001; adjusted model; Table 6). The final overall model predicted 11% of variance in Hcy levels.

## 4. Discussion

The presented findings demonstrate independent and interactive influences of environmental UVR, 25(OH)D levels, and folate variant *MTHFD1*-rs2236225 on Hcy levels in an elderly Australian cohort. These findings are novel, with this being the first study to consider the independent and interactive influences of environmental UVR levels, vitamin D and folate levels and related genetic factors on Hcy levels.

Environmental UVR levels accumulated over six weeks and four months before clinic appointments (i.e., 6W-EDR and 4M-EDR) were independent predictors of Hcy levels. Hcy levels were inversely associated with 4M-EDR previously [11], with Hcy levels further shown to be related to UVR levels over a shorter six week period (6W-EDR) in the current study. Notably, an interaction between 25(OH)D levels and 6W-EDR, but not 4M-EDR, in predicting Hcy levels was shown, indicating an interaction between 25(OH)D levels and a UVR time frame representative of the time-lag between UVR exposure and 25(OH)D changes [52]. When examining the directionality of this interaction, the association between 6W-EDR and Hcy levels was found to be only significant in subjects within the lower quartiles of 25(OH)D status (<33.26 ng/mL). This finding suggests that a relationship between 6W-EDR and 25(OH)D level exists until adequate vitamin D status is achieved. However, the direct effect of 6W-EDR remained a significant independent predictor of Hcy levels in models considering 6W-EDR/25(OH)D interactions, indicating that the relationship between 6W-EDR and Hcy levels is not solely explained by changes in 25(OH)D levels.

Carriage of the *MTHFD1*-rs2236225 polymorphic allele was associated with increased Hcy levels. This finding is supported by a previous investigation finding homozygosity for this variant to be related to increases in plasma Hcy in healthy Mexican American women (*n* = 43) [63]. *MTHFD1* is the gene for the methylenetetrahydrofolate dehydrogenase, cyclohydrolase and formyltetrahydrofolate synthetase 1 (MTHFD1) trifunctional enzyme, which is required for the interconversion of active folate forms 5,10-methylenetetrahydrofolate (THF), 5,10-methenylTHF, and 10-formylTHF [64]. 5,10-methyleneTHF is required for Hcy regulation, being reduced to 5-methylTHF via action of 5,10-methylenetetrahydrofolate reductase (MTHFR), with 5-methylTHF then used as a methyl donor in the remethylation of Hcy into methionine [3]. Murine models of MTHFD1 insufficiency display increased Hcy levels, as well as decreases in methionine and cystathionine, indicative of impaired Hcy regulation [65,66]. The *MTHFD1*-rs2236225 is a missense variant which results in a thermolabile protein with a reduced half-life and synthetase enzyme activity [67]. This variant causes a reduction in 5,10-methyleneTHF formation, which may adversely affect Hcy regulation [68].

The *MTHFD1*-rs2236225 variant was found to interact with 4M-EDR to predict Hcy levels, with the association between 4M-EDR and Hcy levels dependent on the carriage of the polymorphic *MTHFD1*-rs2236225 allele. This association may be explained in part by the thermolability of the *MTHFD1* variant. A previous investigation by the current authors found the association between environmental UVR levels and RBC folate levels to be dependent on the presence of another thermolabile variant: *MTHFR*-rs1801133 [26]. It is possible that the presence of thermolabile enzymes paired with increased UVR exposure leads to compensatory shifts in folate metabolism, which results in Hcy levels being influenced by UVR through a currently unknown mechanism.

Investigations examining the potential relationship between UVR and Hcy status are limited. Prior to studies undertaken by the current group, only two studies had examined Hcy in this context, with potential seasonal variations in Hcy examined, but with no significant findings reported [69,70]. However, these studies examined < 100 subjects residing in Northern European locations and therefore findings may reflect differences in statistical power and environmental UVR levels [69,70]. It is possible that a UVR-related decline in Hcy may only be evident in populations exposed to high levels of environmental UVR, such as Australians [29], with further investigations examining cohorts located in high UVR areas needed. The reported inverse relationship between UVR and Hcy levels should be interpreted with caution, as it may not indicate a benefit of UVR exposure on Hcy metabolism, but an increase in Hcy turnover and/or use in response to increased oxidative stress following UVR exposure. Increased oxidative stress may promote Hcy auto-oxidization and the formation of Hcy into other oxidants not detected by the Hcy assay used here [71,72].

An apparent limitation of this study is that Hcy levels were examined against environmental UVR levels rather than personal UVR exposures. Assessment of personal exposures was not possible due to this being a secondary retrospective cohort analysis. However, given that original subject recruitments and clinic appointments in the RHLS spanned > 18 months (> 80 clinic dates), collective environmental UVR data captured a wide range of UVR levels, with reported findings providing a justification for future investigations using more precise methods. Further strengths of this investigation were that multiple vitamin D and folate variants were examined, with the RHLS cohort well-characterised in respect to several known Hcy determinants, which were corrected for in subsequent analyses. 25(OH)D levels were examined via a HPLC method [33], with HPLC methods shown to be a more precise and sensitive method for 25(OH)D measurement compared to other methods [73]. However, a limitation of this method is that external quality assessment was not obtained via external quality assessment schemes (e.g., via DEQAS). The focus of the current study was to examine these influences in an elderly Australian cohort, given that this sub-population is at heightened risk of UVR-related cellular damage [29,30]. Several novel independent and interactive influences of environmental UVR levels and 25(OH)D levels and folate variant *MTHFD1*-rs2236225 on Hcy levels are presented. However, findings may be age-specific, given age-related decreases in UVR-protection [30], and increases in oxidative stress [74], with further investigation required before findings can be generalised to other populations.

## 5. Conclusions

Accumulated environmental UVR levels and *MTHFD1*-rs2236225 were found to be independent predictors of Hcy status in an elderly Australian cohort. A nutrient–environment interaction between 25(OH)D levels and 6W-EDR was demonstrated, with the association between 6W-EDR and Hcy only significant for subjects in lower quartiles of 25(OH)D status. Furthermore, a gene–environment interaction between *MTHFR*-rs1801133 and 4M-EDR in predicting Hcy was found, with an association between 4M-EDR and Hcy only observed in subjects carrying the polymorphic *MTHFD1*-rs2236225 allele. Presented findings are novel, with further investigation needed to examine if these independent and interactive effects are population or age-specific, and whether they influence the risk of Hcy-related health outcomes.

## Figures and Tables

**Table 1:** Subject Characteristics.

	Male (*n* = 273)			Female (*n* = 346)			All (*n* = 619)		
Continuous Variables	Mean	95% CI	Range	Mean	95% CI	Range	Mean	95% CI	Range
Age	77	76–78	65–93	77	76–78	65–95	77	76–78	65–95
RBC folate (nmol/L)	1625.7	1311.5–1939.8	931.0–2539.0	1342.2	1292.0–1392.5	385.0–2695.0	1340.9	1304.8–1377.1	381.0–2695.0
25(OH)D (ng/mL)	31.5	24.6–38.4	9.7–44.7	35.0	33.3–36.7	9.3–94.4	35.8	34.6–37.0	9.3–94.4
Hcy (μmol/L)	11.3	10.5–12.2	0.1–47.4	10.1	9.5–10.6	0.1–31.2	10.4	9.9–10.9	0.1–47.4
Serum vitamin B_12_ (pmol/L)	224.6	207.4–241.8	12.0–1116.0	251.0	235.6–266.2	68.0–1500.0	239.3	227.8–251.0	12.0–1500.0
Creatinine (μmol/L)	10.6	10.0–11.2	1.4–51.6	8.4	7.8–8.9	0.5–29.5	9.4	9.0–9.8	0.5–51.6
Vitamin B_6_ intake (mg/d)	9.0	4.9–13.1	0.0–220.7	9.2	6.9–11.5	0.0–203.0	8.6	6.9–10.3	0.0–220.7
Alcohol intake (g/day)	10.4	9.0–11.7	0.0–40.4	4.4	3.7–5.1	0.0–37.7	7.0	6.3–7.7	0.0–40.4
6W-EDR	5920.8	5577.9–6263.7	2140.5–11057.4	5959.9	5662.1–6257.6	2140.5–11057.4	6014.4	5718.4–6166.9	2149.5–11057.4
4M-EDR	16795.5	15888.5–17702.5	7788.2–28258.0	16739.0	15939.6–17538.4	7788.2–29160.6	16764.7	16165.8–17361.9	7788.2–29160.6
**Categorical Variables**	***n***	**%**		***n***	**%**		***n***	**%**	
*Tea serves/day*									
<1	78	30		108	34		193	32	
1–2	118	46		141	45		274	46	
>2	61	24		67	21		132	22	
*Coffee serves/day*									
<1	90	35		113	36		208	35	
1–2	112	44		135	43		262	44	
>2	55	21		68	22		129	22	
*Smoking status*									
Current or ex-smoker	185	68		126	36		308	50	
Never smoked	88	32		220	64		311	50	
*BMI category*									
Underweight or normal	49	20		82	26		122	23	
Overweight	121	48		128	40		257	43	
Obese	80	32		107	34		199	34	

Hcy: Homocysteine, 4M-EDR: 4 month accumulated erythemal dose rate, 6W-EDR: 6 week accumulated erythemal dose rate, 2D-EDR: 2 day accumulated erythemal dose rate, BMI: body mass index.

**Table 2:** Associations between accumulated erythemal dose rate (EDR) measures, and levels of 25(OH)D levels, and RBC folate on Hcy levels, with and without adjustments for Hcy confounders.

	Hcy Levels					
	Unadjusted (*n* = 618)		Model 1 (*n* = 579)		Model 2 (*n* = 464)	
	β	*p*	β	*p*	β	*p*
6W-EDR	***−0.25***	***<0.001***	***−0.24***	***<0.001***	***0.24***	***<0.001***
25(OH)D levels	-		0.04	0.3	−0.03	0.5
RBC folate levels	-		−0.02	0.7	−0.03	0.6
4M-EDR	***−0.29***	***<0.001***	***−0.29***	***<0.001***	***−0.30***	***<0.001***
25(OH)D levels	-		−0.05	0.2	−0.05	0.3
RBC folate levels	-		−0.04	0.3	−0.05	0.2

Italics and bold indicate results that are statistically significant. Adjustments: Model 1 = 25(OH)D and RBC folate levels. Model 2 = Model 1 and sex, age, creatinine and vitamin B_12_ levels, reported dietary intake of alcohol, vitamin B_6_, tea and coffee, smoking status and BMI category. Total number of participants in each model vary due to missing data.

**Table 3:** Assessment of interactions between EDR measures (6W-EDR and 4M-EDR) and levels of 25(OH)D or RBC folate in determining Hcy levels, with and without adjustments.

	Hcy Levels			
	Unadjusted		Adjusted	
	β	*p*	β	*p*
6W-EDR	−***0.24***	***<0.001***	−***0.24***	***<0.001***
25(OH)D levels	−0.04	0.3	−0.03	0.5
6W-EDR *x* 25(OH)D levels	***0.10***	***0.01***	***0.15***	***0.002***
*n* = 582/467				
4M-EDR	−***0.27***	***<0.001***	−***0.27***	***<0.001***
25(OH)D levels	−0.05	0.2	−0.03	0.4
4M-EDR *x* 25(OH)D levels	0.04	0.3	0.09	0.05
*n* = 582/467				
6W-EDR	−***0.24***	***<0.001***	−***0.24***	***<0.001***
RBC folate levels	0.00	0.9	−0.01	0.9
6W-EDR *x* RBC folate levels	0.01	0.9	0.01	0.9
*n* = 612/491				
4M-EDR	−***0.29***	***<0.001***	−***0.29***	***<0.001***
RBC folate levels	−0.02	0.6	−0.04	0.4
4M-EDR *x* RBC folate levels	0.01	0.9	0.01	0.9
*n* = 612/491				

Italics and bold indicate results that are statistically significant. *P*-values for interactions were compared against Bonferroni-adjusted *p* thresholds of *p* < 0.008 for 6W-EDR models and *p* < 0.0125 for 4M-EDR models, to account for multiple testing. Tabled variables were entered into models as continuous variables. Adjustments: sex, age, creatinine and vitamin B_12_ levels, reported dietary intake of alcohol, vitamin B_6_, tea and coffee, smoking status, and BMI category. Total number of participants in each model varies due to missing data.

**Table 4:** Association between 6W-EDR and Hcy levels stratified by quartiles of 25(OH)D levels.

	Hcy Levels				
6W-EDR					
by 25(OH)D quartiles (ng/mL)	*n*	β	*p*	mean	95% CI
***Q1 (<23.95)*** Mean: 18.60	107	***−0.35***	***0.001***	10.6	8.6–12.6
***Q2 (23.96–33.25)*** Mean: 28.24	120	***−0.32***	***0.001***	10.2	8.2–12.1
***Q3 (33.26–45.60)*** Mean: 39.51	108	−0.18	0.09	9.8	7.9–11.7
***Q4 (>45.60)*** Mean: 56.88	132	0.01	0.9	10.2	8.3–12.2
6W-EDR *x* 25(OH)D quartiles (as categorical)—p_interaction_ = 0.004					

Italics and bold indicate results that are statistically significant. The *p*-value for interaction between 6W-EDR and 25(OH)D was compared against a Bonferroni-adjusted *p* threshold of *p* < 0.008 to account for multiple testing between 6W-EDR and variables of interest. Values shown are adjusted with sex, age, and Hcy determinants, creatinine and vitamin B_12_ levels, reported dietary intake of alcohol, vitamin B_6_, tea and coffee, smoking status, and BMI category.

**Table 5:** Assessment of gene–environment interactions between *DHFR*-rs70991108 and *MTHFD1*-rs2236225 variants and 2D-EDR or 6W-EDR in determining Hcy levels.

	Hcy Levels			
	Unadjusted		Adjusted	
	β	*p*	β	*p*
6W-EDR	***−0.23***	***<0.001***	***−0.24***	***<0.001***
*DHFR*-rs70991108	−0.06	0.1	−0.03	0.6
6W-EDR *x DHFR*-rs70991108	0.02	0.7	−0.01	0.9
*n* = 611/462				
6W-EDR	***−0.20***	***<0.001***	***−0.17***	***0.001***
*MTHFD1*-rs2236225	−0.05	0.2	−0.09	0.05
6W-EDR *x MTHFD1*-rs2236225	0.06	0.2	0.11	0.05
*n* = 611/461				
4M-EDR	***−0.28***	***<0.001***	***−0.30***	***<0.001***
*DHFR*-rs70991108	−0.06	0.1	−0.03	0.5
4M-EDR *x* *DHFR*-rs70991108	0.02	0.7	−0.01	0.9
*n* = 611/462				
4M-EDR	***−0.23***	***<0.001***	***−0.22***	***<0.001***
*MTHFD1*-rs2236225	−0.05	0.2	***−0.09***	***0.04***
4M-EDR *x* *MTHFD1*-rs2236225	***0.10***	***0.03***	***0.15***	***0.006***
*n* = 611/461				

Italics and bold indicate results that are statistically significant. *P*-values for interactions were compared against Bonferroni-adjusted *p* thresholds of *p* < 0.008 for 6W-EDR models and *p* < 0.0125 for 4M-EDR models, to account for multiple testing. Adjustments: RBC folate levels and Hcy determinants, sex, age, creatinine and vitamin B_12_ levels, reported dietary intake of alcohol, vitamin B_6_, tea and coffee, smoking status and BMI category. Totals shown are for unadjusted and adjusted models respectively. Total number of participants in each model varies due to missing data.

**Table 6:** Independent and interactive influences of EDR measures, 25(OH)D levels and *MTHFD1*-rs2236225 on Hcy levels.

	Hcy Levels			
	Unadjusted (*n* = 578)		Adjusted (*n* = 461)	
	β	*p*	β	*p*
6W-EDR	***−0.20***	***<0.001***	***−0.19***	***0.001***
25(OH)D levels	−0.04	0.3	−0.03	0.6
6W-EDR x 25(OH)D levels	***0.10***	***0.02***	***0.14***	***0.002***
*MTHFD1*-rs2236225	−0.04	0.3	−0.09	0.05
6W-EDR *x* *MTHFD1*-rs2236225	0.06	0.2	0.11	0.05
**Model—R^2^ (*p*)**	***0.07***	***<0.001***	***0.09***	***<0.001***
4M-EDR	***−0.22***	***<0.001***	***−0.21***	***<0.001***
25(OH)D levels	−0.05	0.2	−0.03	0.4
4M-EDR x 25(OH)D levels	0.03	0.4	0.09	0.06
*MTHFD1*-rs2236225	−0.04	0.3	***−0.09***	***0.04***
4M-EDR *x* *MTHFD1*-rs2236225	***0.10***	***0.03***	***0.14***	***0.006***
Model—R^2^ (*p*)	***0.08***	***<0.001***	***0.11***	***<0.001***

Italics and bold indicate results that are statistically significant. Adjustments: RBC folate levels and Hcy determinants, sex, age, creatinine and vitamin B_12_ levels, reported dietary intake of alcohol, vitamin B_6_, tea and coffee, smoking status and BMI category. Totals shown are for unadjusted and adjusted models, respectively. The total number of participants in each model varies due to missing data.

## Supplementary Materials

The following are available online at https://www.mdpi.com/2072-6643/12/5/1455/s1, Figure S1: Binary associations between biochemical variables of interest (Hcy, 25(OH)D and RBC folate levels), Table S1: Allelic and genotypic frequencies for folate and vitamin D variants and assessment for deviation from Hardy–Weinberg equilibrium, Table S2: Folate variants identified by stepwise regression for inclusion in models for Hcy prediction, with and without adjustments, Table S3: Assessment for gene-nutrient interactions in predicting Hcy, with and without adjustments for determinants of Hcy levels.
