# Supplementary material for: Independent and Interactive Influences of Environmental UVR, Vitamin D Levels, and Folate Variant MTHFD1-rs2236225 on Homocysteine Levels
Source: Nutrients. 2020 May 18;12(5):1455. doi: 10.3390/nu12051455 (PMC7284830; doi:10.3390/nu12051455)
Supplement: Supplementary file 1 [file nutrients-12-01455-s001.zip › Supplementary material HCY paper final.docx]

**Supplementary Material**

**Table S1.** Allelic and genotypic frequencies for folate and vitamin D variants and assessment for deviation from Hardy-Weinberg equilibrium

| **Variant** | **Allele***  **n (%)** | | **Genotype**  **n (%)** | | **HWE**  **χ^2^ (p)** |
| --- | --- | --- | --- | --- | --- |
|  |  |  |  |  |  |
| *MTRR*-rs1801394 | Presence of G allele | 489 (79) | AA | 127 (21) | 1.18 (0.3) |
|  | Absence of G allele | 127 (21) | AG | 320 (52) |  |
|  |  |  | GG | 169 (28) |  |
| *MTR*-rs1805087 | Presence of G allele | 220 (36) | AA | 393 (64) | 0.08 (0.8) |
|  | Absence of G allele | 393 (64) | AG | 197 (32) |  |
|  |  |  | GG | 23 (4) |  |
| *MTHFR*- rs1801131 | Presence of C allele | 322 (52) | AA | 292 (47) | 0.00 (0.9) |
|  | Absence of C allele | 292 (48) | AC | 262 (43) |  |
|  |  |  | CC | 60 (10) |  |
| *MTHFR*- rs1801133 | Presence of T allele | 350 (57) | CC | 266 (43) | 3.9 (0.05) |
|  | Absence of T allele | 266 (43) | CT | 294 (48) |  |
|  |  |  | TT | 56 (0.09) |  |
| *TYMS*-rs11280056 | Presence of deletion | 297 (50) | ins/ins | 295 (50) | 0.9 (0.3) |
|  | Absence of deletion | 295 (50) | ins/del | 239 (40) |  |
|  |  |  | del/del | 58 (10) |  |
| *TYMS*- rs45445694 | Presence of 3-repeat | 478 (79) | 2/2 repeat | 129 (21) | 1.36 (0.2) |
|  | Absence of 3-repeat | 129 (21) | 2/3 repeat | 317 (52) |  |
|  |  |  | 3/3 repeat | 161 (27) |  |
| *SHMT*- rs1979277 | Presence of T allele | 320 (52) | CC | 295 (48) | 3.60 (0.06) |
|  | Absence of T allele | 295 (48) | CT | 247 (40) |  |
|  |  |  | TT | 73 (12) |  |
| *RFC1*- rs1051266 | Presence of A allele | 419 (68) | GG | 195 (32) | 0.04 (0.9) |
|  | Absence of A allele | 195 (32) | GA | 300 (49) |  |
|  |  |  | AA | 119 (19) |  |
| *MTHFD1*- rs2236225 | Presence of A allele | 471 (77) | GG | 144 (23) | 0.06 (0.8) |
|  | Absence of A allele | 144 (23) | GA | 304 (50) |  |
|  |  |  | AA | 167 (27) |  |
| *DHFR*- rs70991108 | Presence of deletion | 415 (67) | ins/ins | 200 (32) | 0.28 (0.6) |
|  | Absence of deletion | 200 (33) | ins/del | 307 (50) |  |
|  |  |  | del/del | 108 (18) |  |
| *DHCR7/NADSYN1*-rs12785878 | Presence of T allele | 525 (95) | GG | 27 (5) | 1.03 (0.3) |
|  | Absence of T allele | 27 (5) | GT | 209 (39) |  |
|  |  |  | TT | 316 (57) |  |
| *CYP24A1*-rs17216707 | Presence of C allele | 170 (34) | TT | 334 (66) | 1.17 (0.3) |
|  | Absence of C allele | 334 (66) | CT | 157 (31) |  |
|  |  |  | CC | 13 (3) |  |
| *GC*-rs4588 | Presence of A allele | 286 (51) | CC | 267 (49) | ***5.7 (0.02)*** |
|  | Absence of A allele | 267 (49) | CA | 251 (45) |  |
|  |  |  | AA | 35 (6) |  |
| *CYP2R1*-rs10741657 | Presence of A allele | 338 (63) | GG | 200 (37) | 0.05 (0.8) |
|  | Absence of A allele | 200 (37) | GA | 254 (47) |  |
|  |  |  | AA | 84 (16) |  |
| *VDR*-rs4516035 | Presence of G allele | 413 (67) | AA | 200 (33) | 0.24 (0.6) |
|  | Absence of G allele | 200 (33) | AG | 295 (48) |  |
|  |  |  | GG | 118 (19) |  |
| *VDR*-rs1544410^ | Presence of T allele | 387 (63) | CC (bb) | 223 (36) | 0.09 (0.8) |
|  | Absence of T allele | 223 (37) | CT (Bb) | 294 (48) |  |
|  |  |  | TT (BB) | 93 (15) |  |
| *VDR*-rs757343^ | Presence of T allele | 165 (27) | CC (UU) | 450 (73) | 1.28 (0.6) |
|  | Absence of T allele | 450 (73) | CT (Uu) | 148 (24) |  |
|  |  |  | TT (uu) | 17 (3) |  |
| *VDR*-rs2228570^ | Presence of G allele | 507 (85) | AA (ff) | 91 (15) | 0.49 (0.5) |
|  | Absence of G allele | 91 (15) | GA (Ff) | 295 (49) |  |
|  |  |  | GG (FF) | 212 (35) |  |
| *VDR*-rs731236^ | Presence of G allele | 385 (63) | AA (TT) | 229 (37) | 0.71 (0.4) |
|  | Absence of G allele | 229 (37) | AG (Tt) | 300 (49) |  |
|  |  |  | GG (tt) | 85 (14) |  |
| *VDR*-rs11568820 | Presence of G allele | 533 (96) | AA | 24 (4) | 2.74 (0.1) |
|  | Absence of G allele | 24 (4) | AG | 215 (39) |  |
|  |  |  | GG | 318 (57) |  |
| *VDR*-rs7975232^ | Presence of A allele | 485 (79) | CC (aa) | 129 (21) | 0.97 (0.3) |
|  | Absence of A allele | 129 (21) | CA (Aa) | 318 (52) |  |
|  |  |  | AA (AA) | 167 (27) |  |

*Allelic frequencies reported as presence and absence of polymorphic allele for each variant.

^ VDR genotypes are referenced using base pair notation but are often designated by a lowercase letter and capital letter (e.g. f and F alleles) for the presence and absence of the restriction site respectively in literature – both are given here.


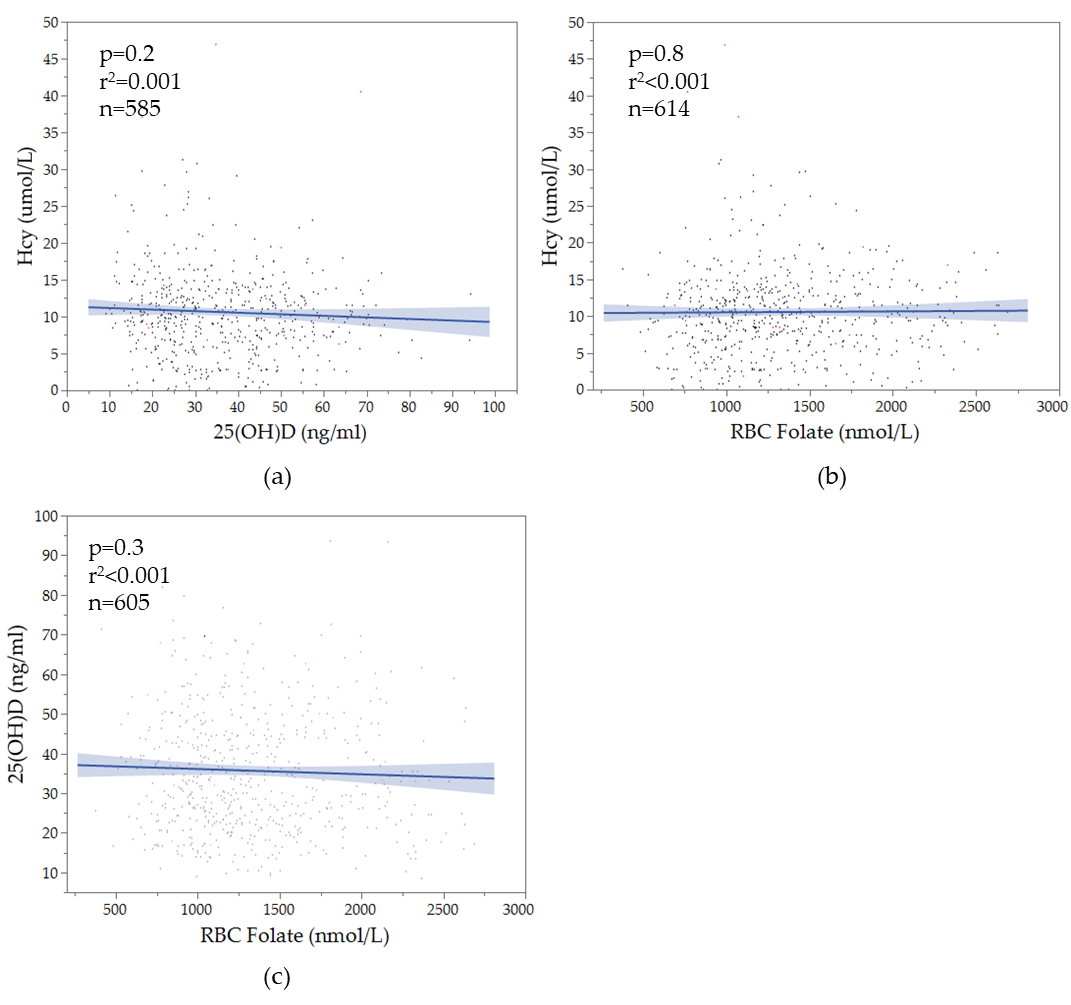


**Figure S1.** Binary associations between biochemical variables of interest (Hcy, 25(OH)D and RBC folate levels).

**Table S2.** Folate variants identified by stepwise regression for inclusion in models for Hcy prediction, with and without adjustments

|  | **Hcy levels** | | |
| --- | --- | --- | --- |
|  | Unadjusted  (n=609) | Model 1  (n=574) | Model 2  (n=460) |
|  | β (p) | β (p) | β (p) |
| *DHFR*- rs70991108 | -0.07 (0.09) | -0.05 (0.2) | -0.03 (0.5) |
| *MTHFD1*-rs2236225 | -0.05 (0.3) | -0.04 (0.4) | -0.09 (0.05) |

Italics and bold indicate results that are statistically significant. Adjustments; Model 1 – RBC folate and 25(OH)D levels. Model 2 – Model 1 and Hcy determinants; sex, age, creatinine and vitamin B_12_ levels, reported dietary intake of alcohol, vitamin B_6_, tea and coffee, smoking status and BMI category. Totals shown are for unadjusted and adjusted models respectively. Total number of participants in each model vary due to missing data.

**Table S3.** Assessment for gene-nutrient interactions in predicting Hcy, with and without adjustments for determinants of Hcy levels.

|  | **Hcy levels** | |
| --- | --- | --- |
|  | Unadjusted | Adjusted |
|  | β (p) | β (p) |
| *DHFR*-rs70991108 | -0.08 (0.06) | -0.07 (0.1) |
| RBC folate levels | 0.05 (0.3) | 0.03 (0.5) |
| *DHFR*-rs70991108 *x* RBC folate levels | 0.03 (0.5) | 0.02 (0.7) |
| n=609/489 |  |  |
|  |  |  |
| *MTHFD1*-rs2236225 | -0.06 (0.1) | ***-0.11 (0.02)*** |
| RBC folate levels | 0.05 (0.3) | 0.04 (0.4) |
| *MTHFD1*-rs2236225 *x* RBC folate levels | 0.02 (0.6) | 0.02 (0.6) |
| n=609/488 |  |  |

Italics and bold indicate results that are statistically significant. P values for interactions were compared against a Bonferroni adjusted p threshold of p<0.025 to account for multiple testing. Adjustments; Hcy determinants; sex, age, creatinine and vitamin B_12_ levels, reported dietary intake of alcohol, vitamin B_6_, tea and coffee, smoking status and BMI category. Totals shown are for unadjusted and adjusted models respectively. Total number of participants in each model vary due to missing data.
